# Mixed dementia: A review of the evidence

**DOI:** 10.1590/1980-57642016dn11-040005

**Published:** 2017

**Authors:** Nilton Custodio, Rosa Montesinos, David Lira, Eder Herrera-Pérez, Yadira Bardales, Lucía Valeriano-Lorenzo

**Affiliations:** 1 Unidad de Diagnóstico de Deterioro Cognitivo y Prevención de Demencia. Instituto Peruano de Neurociencias. Lima, Perú.; 2 Servicio de Neurología. Instituto Peruano de Neurociencias. Lima, Perú.; 3 Servicio de Medicina de Rehabilitación. Instituto Peruano de Neurociencias. Lima, Perú.; 4 Unidad de Diseño y Elaboración de Proyectos de Investigación. Instituto Nacional de Salud del Niño. Lima, Perú.; 5 GESID. Lima, Peru.; 6 Unidad de Geriatría. Instituto Peruano de neurociencias. Lima, Perú.; 7 Unidad de Neuropsicología. Instituto Peruano de Neurociencias. Lima. Perú.

**Keywords:** mixed dementia, Alzheimer's disease, vascular dementia, cerebrovascular disease, demência mista, doença de Alzheimer, demência vascular, doença cerebrovascular

## Abstract

Mixed dementia is the coexistence of Alzheimer's disease and cerebrovascular disease (CVD) in the same demented patient. Currently, its diagnosis and treatment remains a challenge for practitioners. To provide an overview of the epidemiology, pathogenesis, natural history, diagnosis, and therapy of Mixed Vascular-Alzheimer Dementia (MVAD). The literature was reviewed for articles published between 1990-2016 by using the keywords linked to MVAD. Neuropathological studies indicate that MVAD is a very common pathological finding in the elderly with a prevalence about of 22%. The distinction between Alzheimer's dementia and vascular dementia (VD) is complex because their clinical presentation can overlap. There are international criteria for the MVAD diagnosis. The pharmacologic therapy shows modest clinical benefits that are similar among all drugs used in patients with Alzheimer's dementia and VD. The non-pharmacologic therapy includes the rigorous management of cardiovascular risk factors (especially hypertension) and the promotion of a healthy diet. The diagnosis and treatment of MVAD cannot be improved without further studies. Currently available medications provide only modest clinical benefits once a patient has developed MVAD. In subjects at risk, the antihypertensive therapy and healthy diet should be recommend for preventing or slowing the progression of MVAD.

## RESUMO.

Demência mista é denominação usual para a coexistência da doença de Alzheimer e doença cerebrovascular (DCV) no mesmo paciente demente. Atualmente, seu diagnóstico e tratamento continuam sendo um desafio. Fornecer uma visão geral da epidemiologia, patogênese, história natural, diagnóstico e terapia da Demência Mista Alzheimer-Vascular (DMAV). Foi realizada revisão da literatura buscando por artigos publicados entre 1990 e 2016 usando palavras-chave relacionadas ao DMAV. Estudos neuropatológicos indicam que DMAV é um achado patológico muito comum em idosos, com uma prevalência de cerca de 22%. A distinção entre demência de Alzheimer e demência vascular (DV) é complexa porque suas apresentações clínicas podem se sobrepor. Existem critérios internacionais para o diagnóstico DMAV. A terapia farmacológica mostra benefícios clínicos modestos que são semelhantes para todos os medicamentos utilizados em pacientes com demência de Alzheimer e DV. A terapia não-farmacológica inclui o manejo rigoroso dos fatores de risco cardiovascular (especialmente a hipertensão) e a promoção de uma dieta saudável. O diagnóstico e o tratamento do DMAV não podem ser melhorados sem outros estudos. Os medicamentos atualmente disponíveis fornecem apenas benefícios clínicos modestos, depois que DMAV instalou-se. Em indivíduos em risco, a terapia anti-hipertensiva e uma dieta saudável devem ser recomendadas para prevenir ou retardar a progressão da DMAV.

## INTRODUCTION

According to the American Heart Association/American Stroke Association (AHA/ASA), the vascular and neurodegenerative disorders are common in the elderly and it could coexist in the same patient. These processes underlying dementia are mutually potentiated for developing cognitive impairment and dementia, generating overlapped clinical phenotypes and neuroimaging.^1^ Thus, the mixed dementia occurs in patients with a neurodegenerative disorder (such as Alzheimer's disease (AD), Lewy body disease, or Pick body disease) and, additionally, a cerebrovascular disease (CVD).^2^

The mixed dementia, the Alzheimer's dementia, and the vascular dementia (VD) are the most frequent types of dementia,^3^ which could be related to each other. There is evidence of the near linkage between AD and the CVD. For example, CVD lesions such as lacunes and white matter lesions (WML), are common in patients with AD. Likewise, typical pathological changes of AD, as extracellular amyloid plaques (“senile senile”) and intracellular neurofibrillary tangles (NFT), are observed in elderly patients with VD.^4^ Finally, the brain lesions of AD and VD often occur together^5^^-^ 7 and interact in several ways to increase the likelihood of clinically significant cognitive decline.^8^^,^ 9

For an optimal treatment of dementia, the adequate diagnosis of each dementia type is essential. However, recognizing the distinction between these diseases can be difficult in clinical practice. This review provides an overview of the Mixed Vascular-Alzheimer Dementia (MVAD, addressing the epidemiological, pathogenic, clinical, diagnostic and therapeutic aspects of this medical condition.

## METHODS

### Search strategy.

Studies published between 1990 and 2016 were obtained from the following databases: Medline, Biomed Central, Embase, Scopus, Scirus, PsychInFO, LILACS and IBECS. Scholar Google, Ovid, Ebsco, Proquest, Cochrane Library, Cochrane Library Plus and WHO/PAHO databases was reviewed too. All languages were considered. The authors independently searched in specific fields (title, abstract and, if available, subject) for the following terms: (Dementia) AND (“Alzheimer Disease”) AND (“Cerebrovascular Disorders” OR ”Brain Ischemia” OR “Carotid Artery Diseases” OR “Cerebral Small Vessel Diseases” OR “Cerebrovascular Trauma” OR “Intracranial Arterial Diseases” OR “Intracranial Arteriovenous Malformations” OR “Intracranial Embolism and Thrombosis” OR “Intracranial Hemorrhages” OR “Stroke” OR “Central Nervous System Vasculitis”). Abstracts of articles in any language were independently reviewed by the authors. The only inclusion criterion was that the study should be available as full text in any bibliographic or academic databases. We did secondary search of reference lists of identified studies and received additional references from international experts. The latest date of publication for inclusion of articles in the study was 31^th^ July 2017.

### Ethics.

No approval from the ethics committee was required since the study was carried out solely with published data from the world literature.

## EVIDENCE SYNTHESIS

### Selection of studies.

In the consulted databases our search strategy identified 357 scientific documents (original articles, reviews and conference papers), which 26 articles addressed the topic of “mixed dementia” and 8 key articles were selected for this study, including a previous review about mixed dementia.^10^ Additional references were selected through of expert suggestions (3 articles) and secondary search of the mentioned documents. Finally, the total of references of this study were completed according the required information for each section (epidemiology, pathogenesis, etc.).

### Epidemiology.

Several autopsy-based studies reported a dementia prevalence of 39.5%-44.4%,^11^^-^ 13 corresponding 36%-50% to Alzheimer's dementia,^12^^-^ 15 30%-43.0% to DV,^11^^,^ 13 ^,^ 14 and 20%-22% to MVAD.^13^^,^ 16 Other studies have found that 5%,^14^ 28%^17^ and 35%^15^ of the demented subject's autopsies had mixed lesions of CVD-AD. Communities based studies have showed a dementia prevalence of 12.4% (73/590), with a proportion of CVD lesions of 67.1%. In this study the prevalence of MVAD and AD with CVD were 1.7% and 4.7%, respectively.^18^

Other autopsy-based studies showed that the dementia proportion increases from 57% to 75% due to the presence of large artery infarctions and up to 93% due to the presence of lacunar infarcts.^19^ Likewise, the presence of single and multiple infarctions multiplies the dementia risk by 2.12 and 2.67 times, respectively. In autopsies with few neuritic plaques (mature plaques or amyloid plaques) Petrovitch et al. demonstrated that the synergistic effect of CVD-AD through finding a very high proportion of dementia among those with coexisting vascular lesions.^20^

### Pathogenesis.

The MVAD, as well as other dementias, starts several years before the symptoms display, which allow their diagnosis.^21^^,^ 22 The pathogenesis of Alzheimer's dementia is unclear. However, there are two theories that attempt to explain their origin: amyloid theory and vascular theory. Taking into account the theme of this review, we consider pertinent to discuss the last theory.

According to vascular theory, the chronic non-communicable diseases (hypertension, diabetes mellitus, dyslipidemia, obesity, and cardiac disease) and the sedentary lifestyle produce several vascular changes^23^^,^ 24 such as the thickening of the capillary basal membrane and the accumulation of collagen in the vascular endothelium, which both generate vascular atrophy of the vascular terminations, as well as a decrease in the number of terminal blood vessels. These changes affect the cerebral microvasculature and reduces the cerebral blood flow,^9^^,^ 25 which especially observed in untreated hypertensive patients and those treated irregularly or insufficiently.^26^

There have been reports of different mechanisms of vascular damage such as small vessel atherosclerosis, large vessel arteriosclerosis, cortical and subcortical infarcts, lacunar infacts with gray/white matter lesions, WML (leukoaraoisis), demyelination, gliosis, amyloid angiopathy, and small brain hemorrhages.^3^^,^ 27 ^,^ 28 All these damages would progressively accumulate it, producing hypoperfusion, neuronal death and cerebral atrophy, and accumulating beta-amyloid and phosphorylated tau proteins. Considering that neurovascular dysfunction seems to play a key role in the pathogenesis of AD, some researchers have suggested that AD is a primary vascular-cerebral disorder.^29^^,^ 30

According several epidemiological studies, the main vascular risk factors are the age,^31^ hypertension,^32^^,^ 33 hyperhomocysteinemia,^34^^,^ 35 and WML that are shown as hyperintensities (hypersignals) in brain magnetic resonances.^36^^,^ 37 The presence of these factors increases the injury risk to the cerebral blood vessels, leading to CVD, which can progress to cerebral vascular injury (CVI), whether large or small arteries. With the CVD establishment can emerge manifestations of cognitive deficits that exceed those of normal aging, classified as vascular cognitive impairment (VCI), and progressing to affect daily living activities and social/occupational functioning, i.e. VD.^2^^-^ 4

In the case of AD, some studies reported a set of brain changes during the presymptomatic phase, which includes accumulation of beta-amyloid protein, tau protein and phosphorylated tau protein, alterated glucose metabolism,^38^^-^ 40 and cortical thinning^41^ and cortical atrophy,^42^ which occurs 10 years before the onset of symptoms. All these changes have a deleterious and irreversible effect on the brain, and accumulate over time until triggering the clinical manifestations.

### Diagnosis

#### Clinical diagnosis.

There is no consensus for the diagnosis of MVAD. Thus, international referents such as the Alzheimer's Disease Diagnostic and Treatment Centers (ADDTC)^43^ and the National Institute of Neurological Disorders and Stroke and Association Internationale pour la Recherche et l'Enseignement en Neurosciences (NINDS-AIREN)^44^ have proposed diagnostic criteria that differ from each other. A third organization, the Consortium to Establish a Registry for Alzheimer's Disease (CERAD), has not considered MVAD in its classification system.^45^

According to the ADDTC criteria, the diagnosis of MVAD requires the existence of a typical AD and closely dementia related CVD.^43^ The NINDS-AIRRN criteria include evidence of: 1) memory compromise and ³ 2 other cognitive areas; 2) CVI (focal neurological signs and detection of WML in brain images); and 3) dementia onset during the first 3 months after the cerebral stroke.^44^

Additionally, we recommend an approach aimed to identify the dominant component (CVI or AD). Thus, in the majority of patients with predominance of CVI lesions, the initial clinical phenotype should be as VD (i.e., impairment in frontal-executive functions, rather than memory),^46^ with a later deterioration of attention/concentration.^47^ Otherwise, in patients with predominance of AD, the initial clinical phenotype is episodic memory impairment, with a progressively addition of sub-cortical dementia and a step-by-step evolution of disease (which differs with the typical gradual evaluation of the AD).^48^^,^ 49

#### Neuroimaging.

The neuroimaging studies is useful for increasing the diagnostic certainty, particularly in silent lesions such as lacunar infarcts and WML.^47^^,^ 50 However, there are not pathognomonic imaging signs of VCI/VD and the findings should be interpreted based on individual clinical context. According the VASCOG statement, a near temporal relationship between vascular event and the cognitive symptoms/signs must be present to suspect VCI/VD (ideally, 3 months), especially if previous imaging has been undertaken.^47^^,^ 51

#### Pathologic diagnosis.

A definitive diagnosis warrants a neuropathologic verification. Regarding the clinical-pathological diagnosis of MVAD, we suggest to use the recommendations for the AD diagnosis based on morphological changes (Table 1).^52^ According to the guides of the National Institute of Aging (NIA) and the Ronald and Nancy Reagan Institute from the Alzheimer's Association, the postmortem diagnosis of AD can be performed by combining the criteria of CERAD and Braak NFT staging.^53^ Thus, this method allows to estimate the probability that the AD is the cause of dementia through the following rule: low (CERAD = 0-A and Braak = 1-2), medium (CERAD B and Braak 3-4); or high (CERAD C and Braak 5-6).^54^

**Table 1:** Rules for the pathological diagnosis of AD.

• The NIA criteria for assessing plaques (including diffuse and neuritic plaques) in the neocortex and hippocampus per unit and corrected by age.
• The criteria based on the semi-quantitative evaluation of plaques and neurofibrillary tangle in the neocortex and hippocampus.
• The CERAD criteria using the semi-quantitative neuritic plaques count adjusted by age, which along with the clinical history of dementia is used to set up the probability of AD.
• The topographic categorization of neuritic (neurofibrillary) changes, which includes 6 stages: entorhinal (1 and 2), hippocampal (3 and 4), and neocortical (5 and 6).
• The quantitative criteria of the University of Washington, which is a NIA criteria modification.

There are no specific recommendations for the pathological diagnosis of VCI/VD. However, its importance lies in that allows to identify the type of underlying CVD. Additionally, it also allows to identify injuries in cases of non-detectable lesions by current neuroimaging technologies, such as small cystic infarctions, selective neuronal loss, and microinfarcts.

In this context, Jellinger and Atems proposed as a criterion for pathological diagnosis of MVAD the combination of AD (confirmed by autopsy) with presence of multiple lacunar infarcts or CVI lesions located in the cortex, basal ganglia, thalamus, hippocampus and white matter, with an infarcted cerebral volume of 30 - 50 ml, approximately.^52^

#### Diagnosis in investigation studies.

For the research purposes, the AD diagnosis requires the evidence of: 1) amyloid deposition in the autopsy study, low b-amyloid concentrations in cerebrospinal fluid (CSF), or b-amyloid deposition in positron emission tomography; and 2) neuro-degeneration in the autopsy study, high concentrations of phosphorylated Tau in CSF, or pattern of EA atrophy in the cerebral images.^55^^-^ 57 However, amyloid and tau biomarkers are not required in usual clinical practice. On the other hand, the diagnosis of VCI/VD is under discussion due to the heterogeneity of the clinical-neuropsychological phenotype produced by several CVI types. The AHA/ASA recommended establishing a clear causal relationship between the occurrence of vascular event and the onset of cognitive impairment, and between both the location and severity of CVI, clinically detected or identified by brain imaging, with the pattern and severity of cognitive impairment.^1^

#### Diagnostic challenges.

The AHA/ASA highlighted that there are several factors that affect diagnostic accuracy. For example, current imaging technologies are unable to detect microscopic infarctions (≤ 3 mm) and alterations in small vessels such as arteriosclerosis.^1^ Evenly, there are findings in neuroimaging studies that are not pathognomonic of VCI/VD such as microbleeds,^58^^-^ 60 white matter degeneration^1^ and hippocampal atro­phy,^61^^,^ 62 which may also occur in patients with AD.

Cognitive phenotypes. Due to the lack of consensus on the diagnostic criteria and the heterogeneous neuropathological characteristic of the MVAD, this entity has not been widely studied. However, establishing a cognitive profile in relation to AD can be useful for measuring the contribution of CVD/CVI to cognitive impairment and establishing clinical management and therapeutic strategies in patients with MVAD.^28^^,^ 52 ^,^ 63

There are studies showing that the cognitive performance of patients with MVAD is lower than that observed in patients with Alzheimer's dementia,^64^^,^ 65 particularly in the attention,^65^ memory,^66^ denomination^66^ visuoconstruction^65^ and executive functions.^64^ Similarly, in patients with Alzheimer's dementia, the subgroup that developed cerebral infarction showed a greater involvement of memory,^8^^,^ 66 ^,^ 67 language,^67^ denomination,^8^^,^ 66 verbal fluency,^8^ and constructive praxis.^8^^,^ 66

#### Therapeutic guidelines.

Currently, the treatment is basically symptomatic and preventive. The use of acetylcholinesterase inhibitor drugs such as donepezil, galantamine and rivastigmine and antidemential drugs such as memantine can be used in patients with Alzheimer's dementia and VD.^68^^,^ 69

Studies in patients with MVAD have shown a mild improvement, especially in ​​cognition function, with contradictory results in daily living activities and global status, and showing no statistical superiority in the efficacy of either agent.^70^ Considering the potential adverse effects of medication, it is necessary to carefully evaluate its use in these patients.^70^ Memantine, for example, has demonstrated a cognitive effect similar to acetylcholinesterase inhibitors in patients with VD, although with less adverse effects.^71^^,^ 72

Although effective blood pressure control seems to decrease the progression rate of WML in hypertensive patients,^33^ there is no evidence that adequate control of blood pressure or levels of glucose, cholesterol and triglyceride modify the dementia progression.^73^^,^ 74 We suspect that both the early and continue use of these measures could generate the necessary evidence for its inclusion in the clinical protocols.

A healthy diet includes a greater amount of vegetables, fruits, nuts, soy proteins, grains and fish, as well as lower consumption of red meat. Longitudinal studies have shown that this dietary regimens can decrease both the progression rate of cognitive impairment and the risk of developing AD.^75^^,^ 76 Likewise, regular exercise (≥ 30 minutes for ≥ 3 times per week) showed to decrease the progression of cognitive symptoms associated with AD and VD,^77^ improving the independence and maintaining the daily living activities.

Finally, psychological and social support is critical, particularly when the patient is working. This support should also cover the family, who must understand the progressive loss of independence of the patient.

## Conclusions.

There is a public health need for new studies that address the diagnosis and treatment of MVAD. Currently, the available medications offer only a modest clinical benefits once a patient has developed MVAD, without any modification of its evolution. In subjects at risk, the antihypertensive therapy and healthy diet should be both recommend and promoted for preventing or slowing the progression of MVAD.

## Recommendations.

For improving the health outcomes, we recommend thinking in MVAD as probable diagnosis in all demented patients with an atypical presentation of AD and coexistence of risk factors for CVD/CVI.

For improving the value of image studies in the diagnosis of dementia, we recommend to assess the concordance between neuroimaging patterns and postmortem findings.^2^ According the current evidence, it is necessary conduct further studies to develop a better understanding of MVAD.
